# ‘All the progressive forms of life are built up on the attraction of sex’: Development and the social function of the sexual instinct in late 19th- and early 20th-century Western European sexology

**DOI:** 10.1177/09526951231208992

**Published:** 2023-12-06

**Authors:** Kate Fisher, Jana Funke

**Affiliations:** 3286 University of Exeter, UK; 3286 University of Exeter, UK

**Keywords:** development, interdisciplinarity, sexology, sexual instinct, sexuality

## Abstract

This article explores the relationship between sexual science and evolutionary models of human development and progress. It examines the ways in which late 19th- and early 20th-century Western European sexual scientists constructed the sexual instinct as an evolutionary force that not only served a reproductive purpose, but was also pivotal to the social, moral, and cultural development of human societies. Sexual scientists challenged the idea that non-reproductive sexualities were necessarily perverse, pathological, or degenerative by linking sexual desire to the evolution of sociality, often focusing on forms of relationality and care that exceeded biological kinship. As a result, non-reproductive sexual expressions, including homosexual and non-reproductive heterosexual behaviours, were interpreted as manifestations of a sexual instinct operating in the service of human development. These claims were reliant on cross-cultural and historical comparisons of sexual values, behaviours, and customs that rehearsed and reinforced imperial narratives of development premised on racialized, gendered, and classed hierarchies. Sexual scientists mapped diverse sexual behaviours in terms of their perceived evolutionary benefits, contributing to colonial narratives that distinguished between different cultures according to imagined trajectories of development. These contestations around the sexual instinct and its developmental functions played a vital role in allowing sexual science to authorize itself as a field of knowledge that promised to provide expertise required to manage sexual life and secure the global development of human civilization.

In his influential study *Die sexuelle Frage* (The Sexual Question; 1905), Swiss neurologist, psychiatrist, and entomologist Auguste Forel presented the sexual instinct as a vital engine of human evolutionary development. The evolutionary purpose of the sexual instinct was not limited to biological reproduction alone; on the contrary, for Forel, the sexual instinct and its attendant emotions, sensations, fantasies, and thoughts drove far-reaching cultural, social, and intellectual advances: ‘Humanity must hope, for its own sake, that reproduction happens in a way that increasingly enhances its entire physical and psychological traits both in relation to power and physical health as well as in relation to character, reason, will, creative fantasy, optimistic love of work, lust of life, and social feelings of solidarity’ (Forel, 1907[1905]: 4).^1^ Forel was not alone in emphasizing the multifunctional purpose of the sexual instinct, which included, but was not limited to, biological reproduction. On the contrary, as this article argues, sexologists (of varying political outlooks) acknowledged and investigated different functions of the sexual instinct. Their efforts to ascertain which manifestations of the sexual instinct were detrimental or beneficial to imagined processes of human progress and development were central to the emergence of Western sexual science as a field of study in the late 19th and early 20th centuries.^2^

It has long been acknowledged that sexual scientists saw human sexuality as shaped by a powerful sexual instinct or impulse. Yet previous scholarship on psychiatric constructions of the so-called sexual perversions (Davidson, 2001; Nobus and Downing, 2006) did not, in the main, approach instinct as a particularly complex area of sexological theorization. Some scholars have suggested that sexologists viewed instinct in rather basic and uncomplicated terms as a singular drive for reproduction that did not require in-depth exploration. In Arnold Davidson's influential account, for instance, the sexual instinct is presented as purely reproductive and ‘taken as so natural [by sexologists] as not to need explicit statement’ (Davidson, 2001: 15). To support this view, Davidson quotes Austrian psychiatrist Richard von Krafft-Ebing's description of the sexual instinct as manifested in the ‘desires in the consciousness of the individual, which have for their purpose the perpetuation of the species … every expression of it that does not correspond with the purpose of nature – i.e., propagation must be regarded as perverse’ (ibid.). Davidson further argues that this construction of the sexual instinct as a reproductive urge allowed sexologists to classify any and all non-reproductive desires as forms of perversion: sadism, masochism, bestiality, fetishism, and homosexuality were united by their subversion of the ‘natural function of the sexual instinct’ away from ‘propagation’ (ibid.). For Davidson, it was the ‘medico-philosophical conception’ of the reproductive function of the sexual instinct that entailed the pathologization of other sexualities that did not serve the goal of preserving the species or the hereditary transmission of advantageous traits (Nobus and Downing, 2006: 6–7).

Existing scholarship has also demonstrated how this investment in reproductive heterosexuality was fuelled by concerns around evolution and human development. Sexual scientists, such as Krafft-Ebing, scholars argue, saw reproductive heterosexuality as essential to civilizational health and development (Davidson, 2001; Nobus and Downing, 2006), and represented non-reproductive behaviours as motors of social and cultural deterioration that threatened evolutionary development (Gilman, 1985). Scholars have demonstrated how, driven by anxieties about evolutionary decline and degeneration – a broad term used to describe various forms of physical, social, and cultural regression – sexology deployed racist, classist, and ableist ideologies to classify perversions and to champion eugenic forms of reproduction (Bauer, 2017; Härmänmaa and Nissen, 2014; Moore, 2009; Nye, 1991; Pick, 1989; Turda, 2010). However, scholars have also complicated the assumption that sexologists automatically and consistently associated non-procreative sex with deviance, perversion, or degeneration. Evidence from sexological case studies reveals that, when confronted with a diversity of individual expressions of sexual desire, sexual scientists struggled to hold onto neat taxonomic schemes that would allow them to classify behaviours as either healthy or pathological, normal or perverse (Crozier, 2008b; Damousi, Lang, and Sutton, 2019; Doan, 2013; Downing, 2011; Lang, Damousi, and Lewis, 2017; Millard and Callard, 2020; Oosterhuis, 2000). As a result, concepts of the ‘healthy’, ‘normal’, and ‘natural’ were inconsistently applied and widely discussed (Cryle and Stephens, 2017; Doan, 2013).

Considerations of the purpose of the sexual instinct played a central role in explaining the diversity of sexual desire. Recent research on the complexity of sexual scientific constructions of instinct has begun to examine the sexological interest in non-reproductive functions of the sexual impulse (Frederickson, 2014; Leng, 2018). The non-reproductive manifestations of the sexual instinct have received particular attention within scholarship on Freudian psychoanalysis. Freud offered a clear account of the differences between a ‘Geschlechtstrieb’ (object-related, genital, usually reproductive) and a ‘Sexualtrieb’ (non-objectal, not necessarily genital or reproductive) in his 1905 *Drei Abhandlungen zur Sexualtheorie* (Three Essays on the Theory of Sexuality) and in later works. Freud went further than many of his contemporaries in exploring the non-reproductive functions of sexuality and acknowledging that the drive for pleasure can be without any purpose or goal (Davidson, 2001; Katz, 2007; Nobus and Downing, 2006; Sandford, 2018). Yet, as some scholars have noted, Freud was not alone in rejecting the idea that reproduction was the sole purpose of the sexual instinct. Kirsten Leng has demonstrated that contestations around the ‘sex drive’ were central to debates around female sexuality in Germany. More specifically, Leng's work reveals how women sexologists challenged the ‘belief that the sex drive's primary function was reproductive’ and focused attention on the multifaceted elements of the female sex drive (Leng, 2018: 76).

Building on this work, our article argues that sexual scientists’ engagement with the sexual instinct was central to the emergence of sexology as a field of knowledge that claimed authority over the global management of sexuality. We show that explorations of the sexual instinct proceeded firstly from the premise that the sexual instinct was a productive evolutionary mechanism through which humans developed moral, social, creative, and intellectual attributes. Secondly, they were driven by the understanding that these attributes were not secured through reproduction alone. Shared across varied sexological investigations, therefore, was an understanding that the sexual instinct offered a vital mechanism for demonstrating the far-reaching power of sexual desire not only in terms of biological reproduction, but also with regard to the development of society and culture as a whole. If evolutionary instincts shaped sexual practices, desires, and behaviours, and were not simply crude, animal urges (Frederickson, 2014), then the question for sexology became how to account for the global diversity of sexual desires. If non-reproductive sexual desires were not automatically atavistic perversions that hindered human development, then finding functional explanations for the diversity of sexual expression, and understanding the wider benefits of sex beyond reproductive gains, became key objectives. As this article reveals, these questions were at the heart of sexual science, a field driven by efforts to understand the global development of human civilization and intervene in contemporary politics to try and secure what sexual scientists defined as human progress.

## Human development and the social functions of sex

Many sexual scientists understood the exploration of a (variously named) highly complex sexual instinct, drive, or impulse to be the central focus of their work. Not only was it a powerful force governing sexual expression, but the sexual instinct was also constructed as a mechanism through which humans could reach moral, social, creative, and intellectual achievements. In *Psychopathia Sexualis*, Krafft-Ebing presented the sexual impulse as an ‘all conquering, mighty’ force and as ‘the most powerful factor in individual and social existence’, which needed to be studied for the ‘welfare of society’ (von Krafft-Ebing, 1886: 1–2, iv). It was the evolutionary mechanism through which humanity awakened ‘higher, nobler feelings’ that produced societies and cultures capable of creating a ‘world of beauty, sublimity, and morality’ (ibid.: 1). For Krafft-Ebing, the sexual impulse was the strongest driver of moral improvement, which allowed humanity to attain ‘the highest virtues’, since ‘all ethics as well as, perhaps, a good part of aesthetics and religion are ultimately rooted in the existence of sexual feeling’ (ibid.: 2). Similarly, British sex psychologist Havelock Ellis explained that ‘all the progressive forms of life are built up on the attraction of sex’, suggesting that sexual instinct was the force that was responsible for both ‘the physical structure of life’ and ‘its spiritual structure – our social feelings, our morality, our religion, our poetry and art’ (Ellis, 1910: 139). As a result, sexologists saw the analysis of the ‘shifting and multifold phenomena’ involved in expressions of the sexual impulse as one of the most urgent ‘essential problems’ of sexual psychology (Ellis, 1913[1903]: vi–vii).

The sexual instinct assumed such importance in the light of a century of scientific thinking about evolution that had gradually transformed ideas about human instincts.^3^ As Kathleen Frederickson has shown, earlier understandings of instincts as ‘bestial’, unconscious, or intuitive drivers of animal behaviour that needed to be tamed and civilized by human rationality, conscious action, and morality had been modified and supplemented by an understanding of instinct as a purposeful and beneficial force (2014: 15–18). Instincts were understood to be part of the essence of both animal and human behaviour and fundamental to their development (Burnham, 1972; Honeycutt, 2019). The evolutionary models of human instincts that informed sexual science drew especially on Darwinian models of development (Smith, 2013). Darwin challenged the idea that the sex instinct was a primitive force, antithetical to the civilized, by suggesting that social and moral feelings had an ‘instinctive or innate’ basis in lower animals and humans alike (Darwin, 1901[1871]: 150). For Darwin, all social animals were born with an innate faculty for sympathy that then evolved into social instincts and a sense of morality (Hale, 2021; Priest, 2017: 587). He argued that ‘mental charms and virtues’ were advantageous traits that would be chosen via sexual selection and passed onto the next generation (Darwin, 1901[1871]: 944), suggesting that evolutionary processes would favour the development of ever more moral and altruistic communities (ibid.: 933). As a result, from the middle of the 19th century, instincts were often reconceptualized as playing a vital role in human and animal behaviour that was fundamental to their evolutionary development and deployed to explain invented maps of progress that distinguished between more and less civilized societies.

In particular, sexologists mobilized a distinction between reproductive and non-reproductive elements of the sexual instinct to understand how changing sexual behaviours drove the evolutionary development of societies from the so-called primitive to the civilized. The ideas of German gynaecologist Alfred Hegar in his 1894 study *Der Geschlechtstrieb* (The Sex Drive) were especially influential, for instance, on German psychiatrist Albert Moll and Freud. Differentiating between the copulative instinct (‘Begattungstrieb’) and the reproductive instinct (‘Fortpflanzungstrieb’), Hegar argued that it was only among higher (domesticated) animals and cultured humans (‘Kulturmenschen’) that such a distinction was discernible (Hegar, 1894: 1, 3). Hegar was one of many sex researchers who suggested that a purely reproductive instinct tended to fade into the background, becoming less influential in structuring sexual behaviour as people reached ‘higher’ stages of development. According to this argument, the growing detachment between a purely reproductive instinct and a sexual instinct that served additional functions was indicative of civilizational advancement. Progress involved the modification of the sex instinct from one in which individuals sought to gratify a purely reproductive urge, experienced as a selfish lust, to one in which individuals sought to gratify a sexual urge that encompassed the desire to love and care for others.

The wider sexological interest in an evolving sexual instinct sheds light on how scientists framed the broader value of sexual science. In studying the sexual instinct, sexual scientists promised to contribute to explaining the history of human evolutionary development and identifying the changes needed to secure future progress. Manifestations of a changing sexual instinct were marshalled to identify the imagined hierarchies that were evoked to separate different cultures. Going further than Hegar, some sexual scientists suggested that modern developed cultures no longer consciously experienced a reproductive drive. Italian neurologist and anthropologist Paolo Mantegazza (acknowledged by Krafft-Ebing as a key influence) stated that it was not reproduction that ‘impel[led] men and women to unite’; indeed, ‘Propagation is often only an undesired effect of this union for love and pleasure’ (Mantegazza, 1931[1886]: 255). He conceptualized his influential ‘Anthropological Theory of Sex’ in *Gli amori degli uomini* (The Sexual Relations of Mankind, 1886) as a Darwinian study of the ‘infinite variations of [sexual] customs and law’ in an attempt to understand the evolutionary purpose of behaviours that could not be reduced to a reproductive need (Mantegazza, 1931[1886]: 254–5; Taylor and Marino, 2019). British socialist campaigner and writer Edward Carpenter similarly argued that the ‘prime object of Sex’ was ‘physical union’, with ‘generation’ only a ‘secondary … result of this union’ (Carpenter, 1894b: 23–4). Others marshalled the idea of the unconscious to show that individuals were often unaware of the reproductive function of the sexual instinct. For example, German philosopher Eduard von Hartmann's *Philosophie des Unbewussten* (Philosophy of the Unconscious, 1869), which influenced Krafft-Ebing, Freud, and Moll, characterized the sexual drive (‘Geschlechtstrieb’) as an ‘instinct’ that strove for reproduction although the individual was not consciously aware of this reproductive goal (von Hartmann, 1871[1869]: 198). Krafft-Ebing argued that ‘in sexual love, the actual purpose of the instinct, the propagation of the species, does not enter into consciousness’ (von Krafft-Ebing, 1892: 9–10). Moll maintained that the sexual drive (‘Geschlechtstrieb’) arose from the reproductive instinct (‘Fortpflanzungstrieb’), but also stressed that reproduction was not necessarily the conscious aim (‘Zweck’) of the sexual drive (Moll, 1898: 1–8).

Sexologists thus presented scientific investigations of the sexual instinct (and especially its non-reproductive functions) as central to the study of human evolution. As this article shows, contestations around the sexual instinct allowed sexual science to authorize itself as providing the vital expertise required to inform the organization of global societies and encourage future civilizational advance. The field saw the ongoing cultivation and governance of sexual instincts as central to future human development, and thus examined global sexual variation as a means of understanding the purpose of sex. Sexual science promised to identify how the sexual instinct could be managed, at an individual and a social level, to advance and secure the development of the species, nation, culture, and empire.

This understanding of sexual science reveals, as much recent scholarship has established, the centrality of colonial frameworks and fantasies about geographically distanced and often racialized ‘Others’ within sexual scientific research frameworks (Chiang, 2009; see also Bleys, 1996; Hoad, 2000; Leck, 2018; Leng, 2016; Marhoefer, 2022; Schields and Herzog, 2021; Willey, 2018).^4^ As many scholars have shown, the sexological interest in the case study sat alongside an engagement with other inter- and cross-disciplinary forms of knowledge, including comparative evidence of different sexual customs and behaviours from historical, anthropological, sociological, and zoological investigations (Bauer, 2009, 2015; Chiang, 2009; Fisher and Funke, 2015, 2018; Leck, 2016; Leng, 2016, 2018; Moore, 2021).^5^ Cross-cultural and cross-historical investigations of the sexual instinct shifted attention away from the individual case study, placing greater emphasis on human developmental trajectories over large timescales. These forms of evidence took on particular significance because they seemed to offer a window onto the evolution of the function and expression of the sexual instinct over time. Sexologists collected evidence of changing sexual customs and behaviours to chart the evolution of the sexual instinct and to map these onto trajectories of human development, utilizing a hierarchical framework from primitive to civilized. Debates about human evolution, which exercised many sexual scientists, were underpinned by an assumption that the story of civilization, and the requirements of future civilizational progress, were intimately connected to the particular and distinct social and cultural management of sexual behaviours. Understandings of which sexual behaviours were evolutionarily beneficial varied considerably, as we will show below, but they drew on the same sexological logic about the importance of the non-reproductive element of the sexual instinct in shaping human development.

For some, an evolving sexual instinct consisted of the individual moving beyond a purely self-serving gratification of sexual desires and developing a capacity for experiencing love of a sexual partner and children, a process that then extended to the development of social sympathy towards the wider group or society. Bloch identified an advanced, non-reproductive, and evolved sexual instinct in societies organized around conjugal and parental love, presenting the very ability to love a sexual partner and children as well as a wider community as an outcome of evolutionary development. He argued in *Das Sexualleben unserer Zeit in seiner Beziehung zur modernen Kultur* (The Sexual Life of Our Time in Its Relation to Modern Civilization, 1907) that only human beings were capable of experiencing ‘love’ once the purely animal expressions of the sexual instinct had been ennobled to such an extent that reproduction and the preservation of the species were no longer the only goals of sexual expression (Bloch, 1907: 3). This idea drew directly upon the work of French anthropologist Charles Letourneau, whose 1888 *L’évolution du marriage et de la famille* (The Evolution of Marriage and of the Family) was published in English by Ellis as part of the Contemporary Science Series in 1891 and later cited by Bloch (Bloch, 1907: 30–1). Letourneau maintained that the capacity to love was an evolutionary achievement that set ‘higher’ humans apart from ‘lower’ animals, who were focused exclusively on reproduction and for whom sexual desire did not have a spiritual element (Letourneau, 1891: 343–4). This logic was extended to categorize societies that expressed care outside the biological family as yet more developed. French psychiatrist Chares Féré, whose work was read by German sexual scientists like Bloch as a ‘specific study’ of the ‘instinctive element of the sexual impulse’ (Bloch, 1907: 815), suggested that ‘the evolution of sexual instinct shows that the individualistic instincts gradually give way to the social. Social sympathy has its origin in conjugal and family sympathy, which itself is based on parental sympathy and love of children’ (Féré, 1904[1899]: 37). Other key elements of the development of a moral sexual instinct were seen to be the development of modesty, the emergence of monogamy, and the social ‘elevation’ of women.

The narratives of progress and civilizational advancement employed by sexologists often reflected racist and misogynistic understandings of developmental hierarchies (Funke, 2015; Leng, 2016; Willey, 2018). Hegar, and sexual scientists inspired by his work, identified culturally advantageous and civilized expressions of the sexual instinct primarily among white men. Hegar associated a purely reproductive instinct both with women and with racialized populations, who came to represent the allegedly unrefined sexual instinct typical of lower stages of cultural development. Reflecting misogynistic understandings of developmental hierarchies, he elaborated, ‘The typical sexual functions in humans are almost entirely blurred and only menstruation in women, which can be seen as a modified period of heat … can be considered as remnants’ (Hegar, 1894: 3). Sexological accounts of the evolutionary trajectory of the sexual instinct, coupled with cross-cultural and anthropological evidence, were thus marshalled to substantiate hierarchical distance between more and less highly evolved societies and cultures (Willey, 2018: 109). Krafft-Ebing maintained that the majority of non-Western countries represented a backward state of sexual morality, with ‘the Australians, the Polynesians, and the Malays of the Philippines’ revealing the ‘primitive stage’ of development, in which the ‘satisfaction of the sexual needs of humans’ remained ‘like that of animals’ (von Krafft-Ebing, 1886: 2). He suggested that Christian monogamy provided the necessary conditions for men and women to care permanently for each other and for their offspring:

Sexual life represents the strongest factor in individual and social life, the mightiest impulse to use one's powers, acquire property, found a home, and awaken altruistic emotions, first towards a person of the opposite sex, then towards children and, in a broader sense, towards all of human society. (von Krafft-Ebing, 1886: 2)

Here, the notion that Christian monogamy was key to developing social and moral skills, and thereby reaching a higher level of development, embeds Krafft-Ebing's model within a particular body of Eurocentric colonial ideology (Willey, 2018: 102–3).

Other sexual scientists constructed different accounts of human evolutionary development. Some used cross-cultural evidence to critique conventional morality or to find evolutionary benefits in the customs of societies they nonetheless labelled ‘primitive’. For example, some sexologists romanticized supposedly ‘healthy’ expressions of the sexual instinct in the ‘natural’ state of more ‘primitive’ cultures; they argued that Western cultures had taken an evolutionary wrong turn and repressed sexual expressions that were permitted in non-European cultures. Challenging the long-standing association between polygamy and the allegedly primitive and promiscuous selfish gratification of sexual appetites, for instance, Ellis drew inspiration from British surgeon James Hinton in suggesting that a preliminary developmental stage of polygamy had been beneficial for human development (Clark, 2011: 48–9). According to Ellis, polygamy allowed for ‘the great forward step involved in passing from ape to man [which] was associated with a change in sexual habits involving the temporary adoption of a more complex system than monogamy’ (Ellis, 1910: 423). This, Ellis argued, was because a temporary phase of polygamous unions allowed humans to ‘find exercise for the developing intellectual and moral aptitudes, the subtle distinctions and moral restraints’ that are not required within monogamous unions (ibid.). In other words, it was by entering into relationships with multiple partners that moral and social skills could be fostered in a way that would allow for love and care to be extended to individuals beyond the nuclear and reproductive biological family.

The comparative perspectives provided by cross-cultural and cross-historical evidence were used to chart the developmental purposes and perceived benefits of sexual behaviours, allowing sexual scientists to contribute to debates about how sexual lives should be governed and organized across the globe. Reflecting their engagement with broader contemporary narratives of evolutionary development, sexologists assumed that cultures around the globe and the sexual behaviours associated with them could be arranged on a temporal trajectory from the ‘primitive’ past to the more ‘civilised’ present, and they (aligned with fields such as anthropology and ethnography) associated geographical distance with temporal distance (Fabian, 1983). Threaded through sexological writings were assessments of sexual behaviours around the world, which associated some organizations and expressions of sexual desire with civilizational progress or advancement and others with decline or regression. In pursuing this developmental model of instincts, sexual science was committed to colonial and racist constructions that judged societies around the world along an evolutionary trajectory, positioning humans as moving, via manifestations of the sexual instinct, away from a base and natural state associated with animals and so-called ‘lower races’ (Willey, 2018: 107). Moreover, notwithstanding the diversity of sexual scientific accounts of the evolution of the sexual instinct, imperialist knowledges that positioned sexual science as an essential project that should inform the regulation of sexuality across the globe tended to underpin sexology as a whole.

While the sexual politics that shaped Ellis’ sexual science (such as the critique of conventional monogamy) were at odds with those of Krafft-Ebing (and the elevation of monogamy), they both conceptualized instincts as motors of evolutionary change. Although there was little consensus regarding the most beneficial ways of organizing sexual life, sexual scientists agreed that sex played a vital role not just in reproducing the species, but also in facilitating the evolution of moral and social feelings, and used this framework to engage with racialized understandings of human ‘progress’. This rhetoric of progress and development was mobilized to different ends by sexual scientists holding both conservative and reform-oriented or ‘progressive’ political views.^6^ In fact, as Leng (2016) has shown, the very rhetoric of ‘progress’ (and, we would add, ‘development’) was premised on these racist and colonial modes of comparison. Across these different branches of sexology, the increasing separation of sex from the sole purpose of reproduction was used to measure ‘progress’ and establish an increasing distance between more and less highly evolved organisms and cultures (Willey, 2018: 109). This means that sexual science was not always working with a binary separation of reproductive and non-reproductive practices but was looking to make sense of all kinds of sexual behaviours in terms of their developmental benefits.

As a result, examining explorations of the evolutionary functions of the sexual instinct reveals the logic that underpinned sexological investments in mapping sexual variation and that justified their authority not only over diverse reproductive and non-reproductive sexualities, but also over human civilization and sociality as a whole. As we show in the next section, the claim that human progress relied upon the evolution of a non-reproductively focused sexual instinct (motivated by, for instance, love or altruism) allowed some sexual scientists to argue that the most beneficial and highly evolved expressions of sexual desire were entirely detached from an original reproductive purpose. This framework offered sexual scientists a powerful means to defend and champion non-reproductive sexualities as noble and enriching expressions of desire that served not only the individual, but all of society.

## Non-reproductive sex and human development

As the previous section has shown, sexologists maintained that the careful management of sexuality was vital in ensuring civilizational progress and advancement. They saw the sexual instinct as multifunctional, not only serving reproductive purposes, but also driving the development of what they presented as vital moral and social skills. Within this framework, altruism and an understanding of kinship beyond the biological family were markers of civilization and higher development. This meant that non-reproductive sexual practices did not have to be constructed as anti-instinctual, unnatural, perverse, or atavistic, but could also be incorporated into developmental narratives of evolutionary progress embedded in imperialist concerns about modernity, geopolitics, and civilization. These evolutionary models were mobilized to serve different political agendas, as acknowledged above. This section focuses on the ways in which this framework allowed reform-oriented sex researchers in the early 20th century, such as those seeking to repeal laws against homosexuality, involved in free-love movements, or advocating for changes to women's rights, to valorize specific non-reproductive expressions of the sexual instinct by foregrounding their potential for advancing the evolution of society and culture.

A key area in which these arguments were rehearsed were discussions of male homosexuality. Sexual scientists arguing against the criminalization and moral condemnation of male homosexuality sought to champion forms of sexual desire that were not (necessarily) reproductive and that had often been presented as hedonistic and self-centred rather than fulfilling a social and cultural function. As much existing scholarship has shown, reform-oriented sexual scientists often relied upon case studies to demonstrate that individuals who were labelled as, for instance, sexually inverted or homosexual could be highly successful and productive members of society even if they did not reproduce biologically (Crozier, 2008a, 2008b; Oosterhuis, 2000). *Sexual Inversion* (1897), co-authored by Ellis and British historian and poet John Addington Symonds, and the film *Anders als die Andern* (Different From the Others, 1919), co-written by and starring German physician and founder of the Institute of Sexual Science (Institut für Sexualwissenschaft) Magnus Hirschfeld, strategically presented white, middle-class, Western European homosexual men as socially responsible, professionally respectable, and often artistically gifted (Crozier, 2008a; Linge, 2018). This model of what Heike Bauer calls the ‘super invert’ was fed by different intellectual traditions (Bauer, 2009: 127–33), including the Platonic notion that men who desired other men might not reproduce biologically, but excelled at the even more important production of intellectual and artistic works or ‘offspring you might expect a mind to bear’ (Plato, 1998[ca. 385–370 BC]: 52).

When it came to homosexuality, debates about the nature of the sexual instinct, which were not originally focused specifically on same-sex desire, shifted attention away from the question of whether or not an individual's sexual constitution was functionally healthy or normal. They allowed sexual scientists to move on from often pathologizing questions about the reasons why an individual might experience same-sex desire. These considerations also went beyond a eugenic focus on the biological health of offspring – it was not only about ‘breeding’ physically fit children, but about asking how sexual life should be organized to promote social and cultural progress and evolution. In understanding and judging the value and significance of sexual behaviours within society, sexologists could explore the purpose of same-sex behaviours beyond a focus on individual health and reproductive function. Engaging with evolutionary narratives of supposed social and cultural development allowed sexual scientists to look for ways in which same-sex relationships might be implicated in trajectories of human progress, foregrounding the beneficial impact of same-sex sexual behaviours on society and culture over vast developmental time spans.

This approach was central to the German masculinist homophile movement, led by German zoologist Benedict Friedländer and publicist and writer Adolf Brand (Keilson-Lauritz, 2000, 2005). Although scholars have tended to position this group in opposition to sexological constructions of homosexuality, it is more accurate to say that Friedländer drew on his own scientific training to contribute alternative understandings of homosexuality to the emerging field (Fisher and Funke, 2019). He strongly rejected the idea that same-sex desire was due to a combination of male and female traits in a small group of biologically predisposed homosexual individuals, an understanding he traced back to the work of German jurist Karl Heinrich Ulrichs, and which was most famously championed by his colleague Hirschfeld (Friedländer, 1904: xiii). In contrast, Friedländer combined biological and sociological approaches to argue that male-male attraction was a normal and necessary occurrence that could be found among all social animals, including humans. For Friedländer, Brand, and other proponents of this majoritarian, masculinist model, attraction between men was crucial to evolutionary progress, since it enabled social organisms, including humans, to establish bonds and solidarities beyond the biological family and the heterosexual couple (Tobin, 2017). It was only more highly evolved human and non-human animals, it was claimed, that had developed the ability to feel altruistic concern for organisms to whom they were not biologically related.

In fact, Friedländer maintained in *Renaissance des Eros Uranios* (Renaissance of the Uranian Eros, 1904) that love between men and women and between parents and children ‘does not lead to sociality’ and was among the ‘most primitive’ sensations, which humans shared with animals, including highly asocial ones (Friedländer, 1904: 213–14). Drawing on zoological, anthropological, sociological, and historical forms of evidence, Friedländer sought to demonstrate that progress and social development could not be achieved by relying on these base instincts alone. First, as demonstrated by historic societies he understood to embody this ideal, such as ancient Greece or Japan, Friedländer believed that it was only desire and attraction between men, and, in particular, highly virile men, that had allowed humans and other animals to move beyond this ‘primitive' stage and form cohesive and strong social bonds outside an isolated and self-centred family unit. For him, ‘Same-sex love … is identical with the social instinct itself’ (ibid.: 215). Second, Friedländer maintained that bonds created by same-sex love between men were the evolutionary mechanism through which sociality, culture, and morality originated. For Friedländer, increased intellectual powers and cultural productivity could be achieved only through (non-familial) social bonds that transcended a ‘primitive’ reproductive instinct and allowed men to cultivate their ‘higher’ social and aesthetic feelings for other men outside the biological family (ibid.: 214, 222). In other words, it was non-reproductive male-male desire that had played the most vital role in driving developmental progress and allowed humanity to achieve social and cultural excellence.

The masculinist emphasis of this framework demonstrates how gendered hierarchies that had already been embedded in existing evolutionary narratives, as explored in the previous section, reappeared within homophile reform movements. Although Friedländer and Brand were themselves married to women and argued that male same-sex desire was rarely exclusive of heterosexual attraction, they presented desire for women as a ‘primitive’ and socially less valuable form of intimacy. Women were seen not only as incapable of driving social, cultural, and moral progress; they could threaten social stability and advancement by distracting men from their social and cultural duties (Whisnant, 2016: 70). Friedländer also associated women's liberation and heterosexuality with Jewish traditions, which were positioned as antagonistic to a male eros inspired by ancient Greece. Even though Friedländer was Jewish himself, he promoted an antisemitic form of homonationalism in which virile male same-sex bonds and, by extension, social, cultural, and moral progress were associated with the Aryan race (Ilany, 2017). As a result, the model of development promoted by Friedländer, Brand, and followers like Hans Blüher relied on hierarchical developmental narratives of progress and, in turn, inspired misogynist, nationalist, and racist expressions of homophile activism in the German-speaking world.

Although Friedländer was particularly forceful in articulating a model of development in which male-male desire played a vital role, many of his contemporaries explored similar ideas. Indeed, Friedländer was keen to point out that even Hirschfeld sometimes came close to endorsing a majoritarian model that mirrored his own. He pointed to a passage in *Der urnische Mensch* (The Uranian Human Being, 1903) in which Hirschfeld argued that the sexual instinct was never primarily reproductive in any human being; instead, it was aimed mainly at physical and spiritual attraction to other human beings. It was an instinct for sociality (‘Herdentrieb’) that could not be conflated with biological reproduction and was never necessarily limited to opposite-sex desire (Hirschfeld, 1903: 155, cited in Friedländer, 1904: 232). While Hirschfeld did not go as far as Friedländer in foregrounding the privileged role played by same-sex desire in driving developmental progress and establishing sociality, he similarly concluded that homosexual people had often fulfilled their societal purpose and duty by creating ‘values and works for the individual and for the collective’ (Hirschfeld, 1903: 156–8).

Carpenter drew on evolutionary models that positioned the sexual instinct as a driver of social and cultural development to support similar arguments in early works like *Homogenic Love* (1894; Rowbotham, 2009: 190–3). He stressed that the expression of the sexual instinct, including – indeed, especially – homosexual sex (what he called ‘Comrade-love’ or ‘Comradeship’ between men) was not only key to individual health and fulfilment, but also vital to building a strong and cohesive society (Carpenter, 1894a: 3–4, 10). For Carpenter, ‘the arbitrary limitation of the function of love to child-breeding’ meant that the many social benefits and values of same-sex desire had been overlooked and denied (ibid.: 33). He suggested that modern European life was impoverished due to ‘the fact that the *only* form of love and love-union that it has recognised has been one founded on the quite necessary but comparatively materialistic basis of matrimonial sex-intercourse and child-breeding’ (ibid.: 45; emphasis in original). To remedy this situation and allow for the flourishing of ‘the higher and spiritual life of a nation’, homogenic love needed to be accepted and championed (ibid.: 45).

Carpenter's argument relied on modelling the history of human societies along evolutionary trajectories. For Carpenter, historical examples provided powerful illustrations of the role of same-sex attachments in the evolutionary development of past societies across the globe. The ‘highest manifestations of life’, such as ‘sacrifice’, ‘unswerving devotion’, and ‘life-long union’, were enthusiastically and heroically adopted among many societies, such as the ‘post-Homeric Greeks’ or the ‘Polynesian Islanders’, in ways that were not possible for those preoccupied with heterosexual marriage and child-rearing: ‘It is not to be expected (though it may of course happen) that the man or woman who have dedicated themselves to each other and to the family life should leave the care of their children and the work they have to do at home in order to perform social duties of a remote and less obvious, though may-be more arduous, character’ (Carpenter, 1894a: 4–6, 43–4).

Although a shared militarist and nationalist rhetoric cuts across many of the historical examples chosen by Friedländer and Carpenter (Oosterhuis, 1992), the latter was more invested in a Whitmanian notion of democracy. Like other contemporaries, he felt that homogenic love could lead to renewed bonds between ‘members of different classes’ (Carpenter, 1894a: 47), thereby enabling the ‘building up of new forms of society, new orders of thought, and new institutions of human solidarity’ (ibid.: 45; M. Cook, 2003: 122–42; Funke, 2013; Taddeo, 2002: 15–50). To develop this argument, Carpenter mapped the achievements of homosexual love against evolutionary paths that distinguished ‘civilized nations’ from ‘savage races’, and which saw the evolution of the sexual instinct as a journey from the expression of ‘animal desires’ to altruistic desires and higher sentiments that took civilization from the allegedly primitive to the civilized, cultured, and egalitarian:

Even among savage races lower down … in the scale of evolution, and who are generally accused of being governed in their love-relations only by the most animal desires, we find a genuine sentiment of comradeship beginning to assert itself – as among the Balonda and other African tribes, where regular ceremonies of the betrothal of comrades take place. (Carpenter, 1894a: 7)

As this quote demonstrates, even though Carpenter was invested in tracing valuable expressions of homosexual comradeship across cultural contexts, his argument nevertheless continued to rely on hierarchical racialized distinctions between the primitive and savage, and the cultured and civilized.

Carpenter's later works elaborated the idea that individuals who did not reproduce biologically deployed their sexual passions in the service of civilizational development. In works like *The Intermediate Sex* (1908; versions of which had appeared in 1897), ‘On the Connection Between Homosexuality and Divination’ (1910), and *Intermediate Types Among Primitive Folk* (1914), he complemented the masculinist focus of his earlier approach to argue that individuals belonging to the ‘intermediate sex’ could play a vital role in evolutionary processes precisely because they combined male and female traits – an idea Friedländer, who was focused exclusively on highly virile men, would have rejected vehemently. In an argument rejecting the pathologization of ‘intermediate types’, Carpenter described the ‘special nature of their love sentiment’ as a set of exceptional instinctual capacities. As he explained, the ‘male of this class’ had an ‘instinctive artistic nature’, a ‘sensitive spirit’, a ‘wavelike emotional temperament’, and a ‘hardihood of intellect and body’, whereas the female intermediate had ‘masculine independence’ and ‘strength’ combined with ‘feminine grace’. Societies depended on the ‘command of life’ provided by intermediates’ ‘double nature’ (Carpenter, 1921[1908]: 37–8). Indeed, Carpenter argued, evolution itself was driven by sexual intermediate types who, freed from the burdens of reproduction, could devote more energy to the activities and preoccupations that led to the innovations that constituted civilizational progress. Unencumbered by the gendered distribution of labour between male warriors or hunters and female domestic or agricultural workers, they drove civilization via cultural invention:

Had it not been for the emergence of intermediate types – the more or less feminine man and similarly the more or less masculine woman – social life might never have advanced beyond these primitive phases.… They became inventors and teachers of arts and crafts, or wizards (as they would be considered) and sorcerers; they became diviners and seers, or revealers of the gods and religion; they became medicine-men and healers, prophets and prophetesses, and so ultimately laid the foundation of the priesthood, and of science, literature and art. Thus … it was primarily a variation in the intimate sex-nature of the human being which led to these important differentiations in his social life and external activities. (Carpenter, 1910: 234)

By creating the intellectual and spiritual foundations of culture and providing new tools for understanding and communication, sexual intermediate types played a vital role in driving social and cultural development (Dixon, 1997: 412–13). Carpenter even speculated that some intermediate individuals would have the gift of an alternative order of perception he called the ‘cosmic consciousness’ (Carpenter, 1910: 236), which would allow them to become one with nature and heal the divisions of the modern civilized world.

Such debates about the role of non-reproductive sexual behaviours in furthering evolutionary development were not limited to discussions of male homosexuality. Similar arguments were also mobilized when exploring relations between men and women and featured prominently in debates about marital intimacy, family limitation, and birth control and contraception.^7^ Alongside eugenic strands in population debates that were focused on biological reproduction sat a more general understanding that the sex instinct needed to be managed to bring about additional evolutionary benefits. Sexual scientists were part of the coalition of economists, eugenicists, sociologists, feminists, and socialists who were concerned about global population trends, national health, and gender and sexual relationships (Bashford, 2007). These voices, which could be conservative or reformist, secular or religious, gained momentum during the last decades of the 19th century. By the 1920s, an identifiable birth control movement set out to shape governmental policies and sought to provide advice to men and women about the benefits of controlling reproduction (Grossmann, 1995; MacNamara, 2018; Rusterholz, 2018; Soloway, 1982). Sexological organizations and networks, including the British Society for the Study of Sex Psychology, the World League for Sexual Reform, and the Institute of Sexology in Berlin, actively participated in conversations around birth rates, including discussions about whether and how the numbers of births might be managed to the advantage of both the individual couple and society as a whole (Matte, 2005). These debates about reproductive control drew directly and explicitly on sexological frames of reference and theorizations of the sexual instinct, and in turn linked the politics of reproduction, contraception, and population control to racialized assumptions about the evolutionary development of civilization (Carey, 2012; Dickinson, 2007; Garton, 2004: 161–7; Kline, 2005; Leng, 2018).

In addition, debates about reproductive control and evolutionary development drew on specifically gendered assumptions that male and female instincts followed different evolutionary trajectories and were governed by different logics. Although discussions of the sexual instinct were often universalizing and assumed a male actor, sex researchers nonetheless differentiated between male and female sexual instincts, often deploying arguments that were refracted through ideological frameworks that located advanced instinctual sexual expressions with masculinity, whiteness, middle- and upper-class status, and Christianity, to name only a few categories, and/or assumed that women hindered development by expressing irrational, overemotional, or otherwise ‘primitive’ stages of development. Reform-oriented sexologists writing at the beginning of the 20th century often highlighted the intensity of female sexual impulses, in contrast to those (such as Forel, Krafft-Ebing, and, more cautiously, Moll) who saw women as having a strong maternal drive and comparatively weak sexual desires (von Krafft-Ebing, 1886: 10; Forel, 1907: 93; Moll, 1898: 5–6).^8^ In contrast, Ellis suggested that this misunderstanding of women's sexual instinct was a result of inadequate male lovemaking, and Bloch drew upon his conversations with ‘a great many educated women’ to claim that women's ‘sexual sensibility’ was as intense and enduring as men's (Bloch, 1907: 89; Ellis, 1913[1903]: 50–7). For some female sex researchers, as Leng has illustrated, a specifically female sex drive could be harnessed to establish a progressive role for female sexual subjectivity apart from maternity or reproduction (Leng, 2018: 68).

Here, too, sexual expression was the mechanism through which ‘higher’ (often especially human) developments, including spirituality, creativity, morality, altruism, love, and social cohesion, were achieved in ways that opened up discussions of non-reproductive sex and its benefits. A diverse range of sexual scientists and reformers were united in their belief that non-reproductive sex between men and women could improve intimate bonds, allow individuals (and especially women) to realize their full potential in life, and, finally, lead to a more cohesive and strong society by fostering social, moral, and intellectual skills (Jones, 2023; Laipson, 1996; Sears, 1977). In these early 20th-century debates, the initially tentative teasing apart of different-gender desire and biological reproduction discussed in the previous section led to a more explicit articulation of the individual and social advantages of non-reproductive sex between men and women that would become central to modern constructions of heterosexuality (H. Cook, 2004). These writings expanded understandings of male-female relationships and intimacies beyond their reproductive function while also remaining invested in hierarchical narratives of development and civilizational advancement that were reliant on the racist, colonial, classist, gendered, and ableist frameworks mentioned above.

Some sexologists presented the achievement of social and moral feelings of altruism and care as an outcome of civilization that marked an allegedly ‘higher’ stage of evolution setting predominantly white Western European societies apart from allegedly more primitive animal and human cultures around the globe. For instance, British psychoanalyst Jessie Margaret Murray, who wrote the preface to British palaeobotanist and birth control campaigner Marie Stopes’ 1918 *Married Love*, insisted that ‘the age-long conflict between the “lower” and the “higher” impulses, between the primitive animal nature and the specifically human developments of an altruistic and ethical order, are fought afresh in each soul and in every marriage’ (Murray, quoted in Stopes, 1919[1918]: ix). In *Married Love*, Stopes associated the development of the social values of altruism and ethics with non-reproductive sex between men and women within marriage. Although she considered procreation between ‘healthy’ married couples as ‘the supreme purpose of nature’ (Stopes, 1919[1918]: 78), she nevertheless insisted that pregnancies had to be well timed and limited. This was important to ensure the health of the child, but also to preserve the intimate bond between the married couple, which could otherwise be disrupted by ‘the inevitable dislocation and readjustment necessitated by the wife's pregnancy and the birth of a child’ (ibid.: 81). To experience ‘the mutual glow of rapture in their sex-union’ (ibid.: 82), Stopes suggested, couples required training in the techniques of lovemaking and freedom from the fear of an unplanned pregnancy. This was not only of benefit to the individual couple and their children, but also central to a harmonious and progressive society, given its perceived structural dependence on the stability of the heterosexual monogamous unit. Here, non-reproductive sex was seen to benefit not only the couple but also society as a whole, and presented as a marker of civilization. Stopes' widely discussed eugenic investment in classist, ableist, and racist ideologies was not only premised on notions of physical fitness, but also linked to her conviction that only some individuals were able to practise altruistic self-restraint and control for a greater social good. For Stopes, ‘the racially negligent, the thriftless, the careless, the very lowest and worst members of the community’ were incapable of harnessing the non-reproductive power of the sexual instinct to benefit society (Stopes, 1921: 236).

This framing of the ‘higher’ sexual instinct as associated with altruistic and ethical ideals was echoed in Theodoor van de Velde's influential *Ideal Marriage* (1926). A Dutch author of tens of works on gynaecology and obstetrics, van de Velde highlighted the significant individual and social benefits of marital sex beyond procreation, which, he argued, needed to be regulated via control of reproduction. He maintained that marriage ‘gives the fullest opportunity for the primitive urges, which are initially purely self-regarding and self-centred, to extend themselves in action and consciousness towards *altruistic* objects, i.e., the preservation and welfare of other persons’ (van de Velde, 1933[1926]: 17; emphasis in original). Non-reproductive sex was an essential element of the ‘ideal marriage’ that represented the culmination of the sexual instinct from a ‘simple urge to reproduce’ into a ‘highly evolved love’ (ibid.: 20, 17). This, van de Velde maintained, was a unique achievement of the so-called ‘civilised races’, for whom ‘the urge to reproduce has ceased to “play lead” among the components of the sexual impulse, which appears as a further stage of evolution; an advance in psychic power and complexity’ (ibid.: 11). In this way, the social and individual benefits of non-reproductive heterosexual sex were contrasted with notions of the primitive that, as noted above, were frequently racialized and classed.

At other times, sexual scientists inverted this linear narrative of development, blaming civilization for alienating individuals from the supposedly natural ability to perceive and respect their partner's sexual needs. Here, the fact that heterosexual life was not limited to procreation, but also involved pleasure as well as altruism and care, was presented as a universal and timeless truth that modern society had to rediscover. Ellis, for instance, stressed that sexual intercourse detached from procreation had a ‘full right to existence. In its finer manifestations as an art it is required for the full development of the individual, and it is equally required for that stability of relationships which is nearly everywhere regarded as a demand of social morality’ (Ellis, 1910: 576–7). When elaborating on what he called the ‘art of love’, he further explained that man's sensitivity towards women's sexual needs was not an achievement of civilization, but rather ‘universal in the sexual relationships of the animals below man; it is only at the furthest remove from the “brutes”, among civilised men, that sexual “brutality” is at all common’ (ibid.: 540). Moreover, Ellis stressed that cultures he described as ‘primitive’ regularly taught ‘the art of love’ as part of sexual initiation protocols (ibid.: 507, 515–16), a practice that, he explained, had suffered from centuries of repressive Christian morality in Britain and other Christian countries. Paradoxically, Stopes, too, argued that modern technologies of birth control and sex advice were needed to allow individuals to rediscover the allegedly primitive and natural instinct to sense and respect their partner's sexual needs and rhythms, which had supposedly ‘died out’ over the course of civilization (Stopes, 1919[1918]: 110).

In addition to enhancing the quality of marital intimacy, some sex researchers saw non-reproductive sex as critical in enabling individuals to realize their full potential as creative, purposeful, moral, and intelligent beings that could be of service to society and evolutionary development. Ellis, citing both Bloch and Carpenter, stressed the value of sexual love that was about ‘spiritual creation’ rather than procreation (Ellis, 1910: 510). For American gynaecologist and birth control pioneer Alice Bunker Stockham (Silberman, 2009), ‘Copulation is more than a propagative act; it is a blending of body, soul and spirit.… For both husband and wife, it has a function in soul development’ (Stockham, 1903[1896]: vii). This view was repeated by Carpenter, who quoted directly from Stockham's work in the 1906 reworking of *Love's Coming-of-Age*, to champion deliberately non-reproductive sexual intercourse as resulting in a ‘complete *soul union,* a strange and intoxicating exchange of life, and transmutation’ (Carpenter, 1915[1906]: 173; emphasis in original; Rowbotham, 2009: 281, 342–3).

The argument that non-reproductive sex facilitated new forms of intellectual and spiritual growth and social service was particularly potent for women, positioned as a necessary step in the refinement of women's sexual expression and their ability to contribute to civilizational advance. Some reformers argued that the burdens of excessive reproduction restricted women's ability to develop their creative and intellectual abilities. Carpenter, for instance, suggested that releasing women from constant childbearing would allow them to be more active artistically and creatively, leading to ‘a coming period of considerable artistic expansion and development’ (Carpenter, 1927: 130–6). The emphasis on voluntary motherhood did not mean that feminist and/or sexual scientific writers wanted to deny the importance of motherhood and the maternal instinct. On the contrary, the argument that women were destined to be ‘mothers of the race’ allegedly made them more altruistic than men and gave them the time and opportunities to exercise this influence to benefit society and its development. Thus, elements of women's sexual desires that were constructed as specifically female, including maternal instincts, continued to be seen as having a particularly ennobling effect on female sexuality that, some suggested, needed to be harnessed for the wider social good by limiting (rather than eradicating) motherhood (Leng, 2018: 93). Alongside ‘the generation of physical offspring’ women would, as Stockham explained, also contribute to another ‘department of procreation’; they could beget ‘new inventions’, ‘new thoughts’, and ‘grand conceptions of the true, the beautiful’ or ‘the useful’ (Stockham, 1903[1896]: 89–100). Stockham argued further that the ‘brooding care of the female’ was not only beneficial for motherhood alone, but also allowed women to play valuable roles ‘in our government, in our religious and educational institutions, in all the affairs of life’ (ibid.: 52). Ellis similarly stressed that married mothers needed to be freed up from the demands of frequent childbirth to contribute to professions that apparently demanded maternal experience, such as teaching (Ellis, 1910: 588). In this articulation, the knowledge and technologies required to control reproduction would allow women to become leaders of cultural and social development not only through the production of biological children, but also by harnessing the socially valuable qualities associated with their sexual and maternal instincts.

These arguments in favour of women's rights to make reproductive choices often continued to be underpinned by exclusionary narratives of respectable citizenship and civilizational development. This is forcefully demonstrated in Leng's study of the radical feminist journals *Mutterschutz* (Protection of Mothers) and *Die neue Generation* (The New Generation). These journals – edited by German campaigner Helene Stöcker from 1905 to 1933 – served as the official publication of Germany's League for the Protection of Mothers and Sexual Reform (Bund für Mutterschutz und Sexualreform), which was founded by Stöcker and included Bloch, Forel, Hegar, and Hirschfeld as members (Leng, 2018: 197–202). The League defended the rights of mothers (whether they were married or not) and their children while also advocating for the legalization of contraceptives and abortion. The journals repeatedly ‘drew contrasts with cultural and racial Others who supposedly represented humanity's sexual past’ to bolster reform-oriented arguments, including the claim that ‘(hetero)sexual independence for women’ constituted the ‘next stage in the unfolding of evolutionary law’ (Leng, 2016: 64, 65). This is evident in Stöcker's programmatic ‘On the Reform of Sexual Ethics’, published in the first issue of *Mutterschutz* in 1905. The article put emphasis on the far-reaching importance of the sexual instinct (‘Geschlechtstrieb’) in allowing ‘men and women to strive for a new, higher development together’ (Stöcker, 1905: 12). Stöcker rejected the idea that sex was ‘moral’ only if it served a procreative aim as ‘hypocritical’ (ibid.: 9) and argued that women, in particular, needed to have more control over their sexual partnerships and reproductive decisions. Stöcker's acknowledgment of the non-reproductive functions of the sexual instinct was strongly shaped by eugenic anxieties about allegedly undesirable forms of reproduction. These concerns sat alongside a celebration of the sexual instinct as having the power to open up more nuanced forms of ‘comradeship, friendship and love’ between men and women that would not only make them better parents, but also lead to more advanced forms of heterosexual intimacy and social cohesion (ibid.: 6). This material (again) reveals the reliance by sexual reform movements seeking to valorize non-reproductive expressions of the sexual instinct (alongside attempts to control its procreative potential) upon temporal and cultural distinctions that excluded individuals and groups who were, for various reasons, deemed backward or primitive from contributing to social and cultural development.

Overall, our article has shown that late 19th- and early 20th-century sexual science was reliant on evolutionary frameworks and developmental narratives that presented the sexual instinct as complex, multifunctional, and more than a reproductive drive. Examining sexual scientific debates about the sexual instinct and its far-reaching role within social and cultural development draws attention to the ways in which sexual science authorized itself as a cross-disciplinary field of knowledge promising to provide vital expertise to influence the management of sexual life and secure the global development of human civilization. Sexologists with different political investments maintained that non-reproductive expressions of the sexual instinct could drive cultural, social, and economic advancement. Reform-oriented sexual scientists used this argument to valorize non-reproductive sexualities, thus appropriating the very same hierarchical and exclusionary frameworks of evolutionary development that were also mobilized by their more conservative colleagues. As a whole, this article draws attention to the ways in which focusing on constructions of the sexual instinct within narratives of development can expand existing understandings of the emergence of the sexual scientific field, its preoccupations, and its politics.

## Notes

This article is jointly and equally researched and written. Fisher and Funke should be acknowledged as joint first authors.

1. All translations of German sources are the authors’ own.

2. Scholars generally agree that, from around the middle of the 19th century, a diverse body of research emerged that had a fundamental impact on modern attitudes to sex, gender, and sexuality. While this body of research is often labelled as ‘sexology’, historical understandings of what this field was, and who counts as a sexologist, are contested. Our position is that 19th- and early 20th-century sexual science lacked clear disciplinary boundaries, neatly defined terms of enquiry, and firm institutional structures. As a result, sexual science is best viewed as comprising an eclectic body of work. Various scientific fields contributed to sexual scientific understanding, and evidence was drawn widely, including from biology, anthropology and ethnography, history, criminology, psychology, embryology, gynaecology, and endocrinology. Sexual science also encompassed various (often intersecting) political and ideological commitments. In this article, we use the term *sexologist* or *sexual scientist* to refer to this capacious group of sex researchers; however, as part of acknowledging the flexibility of the contours of the field as it emerged, and to address the contestations that existed (and continue to exist) about what is and what is not legitimate scientific knowledge about sex, we simultaneously describe sexologists by their other professional and political positions (as, for instance, psychiatrists or feminists; Leng, 2018). Sexual science was also a global field of knowledge, fully embedded in the imperial structures of knowledge production that dominated patterns of global exchange and interaction in the 19th and early 20th centuries (Chiang, 2009; Fuechtner, Haynes, and Jones, 2018). For the purposes of this article, we are concentrating on European networks of sexological knowledge with a particular focus on material written in English and German.

3. The concept of instincts shaping human behaviour has a long history, but, before the middle of the 19th century, *instinct* was a rather vague term used to capture non-rational and intuitive impulses. The second half of the 19th century saw increased debate and concern about how to characterize and classify instincts, which, for the purposes of this article, were notable in reconceptualizing instincts as key to evolutionary processes (Frederickson, 2014). As several scholars have noted (Frederickson, 2014: 194; Leng, 2018: 65; Van Haute, 2017), the historical sources use a variety of terms (‘drive’, ‘impulse’, ‘instinct’), sometimes without differentiating between them, sometimes applying their own definitions, and sometimes employing their own particular terminology. There are additional complications when considering how related terms, such as the German *Trieb* and *Instinkt*, were translated into English.

4. This article modifies the idea that sexology took an anthropological or global turn at the beginning of the 20th century (Chiang, 2009; Fisher and Funke, 2018; Leck, 2016). It builds on Howard Chiang’s important work, but demonstrates that – well before the alleged anthropological turn often associated with the work of Iwan Bloch – anthropologists, psychiatrists, and other scientists shared a fascination with the role sex, specifically opposite-sex desire, played in shaping the trajectory of human development and civilization. This means that a process of ‘scientification’, to use Chiang’s helpful terminology, was already taking place earlier in the 19th century, even though interdisciplinary exchange was far less formalized or systematic than it was in the early 20th century.

5. As several scholars have acknowledged, Western sexual scientists at the turn of the 20th century explicitly and determinedly identified sexology as a cross-disciplinary field of knowledge (Bauer, 2015; Fisher and Funke, 2015; Leng, 2018; Leng and Sutton, 2021). Existing accounts of the history of sexology suggest that this interdisciplinary agenda was the result of a broadening out from an earlier, primarily psychiatric, starting point of sexological knowledge production. Our argument in this article is that the strands of knowledge that produced sexual science always drew on a range of methodological approaches and forms of knowledge. This explains why sexology, as it took institutional shape via the creation of organizations, journals, congresses, and national and international networks, did so as an intellectually capacious endeavour, combining insights from (variously entangled) psychiatric and forensic as well as anthropological, sociological, evolutionary, historical, and other fields of knowledge.

6. Scholars who have differentiated between reform-oriented and conservative sexology include Leck (2016: 46–7), Mancini (2010), and Matte (2005). Scholarship that has drawn connections between these branches of sexology includes Sigusch (2008: 198–233).

7. We use *non-reproductive sex* to encompass any sexual regime that sought to prevent (or reduce the chances of) conception, including, for example, individuals restricting the frequency of sexual intercourse, seeking to control the timing of intercourse, engaging in non-reproductive sex acts, or employing contraceptive appliances.

8. The existence of a maternal drive was much contested. See, e.g., Leng (2018: 66–7).
